# Road Anomalies Detection System Evaluation

**DOI:** 10.3390/s18071984

**Published:** 2018-06-21

**Authors:** Nuno Silva, Vaibhav Shah, João Soares, Helena Rodrigues

**Affiliations:** Information Systems Department, University of Minho, 4800-058 Guimarães, Portugal; shah@dsi.uminho.pt (V.S.); joao.soares@dsi.uminho.pt (J.S.); helena@dsi.uminho.pt (H.R.)

**Keywords:** road anomalies, PCA, Fi-Ware, data-mining, collaborative mobile sensing

## Abstract

Anomalies on road pavement cause discomfort to drivers and passengers, and may cause mechanical failure or even accidents. Governments spend millions of Euros every year on road maintenance, often causing traffic jams and congestion on urban roads on a daily basis. This paper analyses the difference between the deployment of a road anomalies detection and identification system in a “conditioned” and a real world setup, where the system performed worse compared to the “conditioned” setup. It also presents a system performance analysis based on the analysis of the training data sets; on the analysis of the attributes complexity, through the application of PCA techniques; and on the analysis of the attributes in the context of each anomaly type, using acceleration standard deviation attributes to observe how different anomalies classes are distributed in the Cartesian coordinates system. Overall, in this paper, we describe the main insights on road anomalies detection challenges to support the design and deployment of a new iteration of our system towards the deployment of a road anomaly detection service to provide information about roads condition to drivers and government entities.

## 1. Introduction

With the increase in number of vehicles on the roads and number of road networks, the issues related to road safety have become more pressing and complex. According to a World Health Organisations report, about 1.25 million people died in road accidents in 2015 [1]. Looking to data provided by Eurostat [2], only in the European Union, car accidents killed more than 25,000 people in 2016. Although Mohammed et al. [3] did not find a direct impact of poor road condition on the number of accidents or accident related deaths, Teschke et al. [4], Ahlin et al. [5] showed that driving on roads with poor conditions negatively impacts the health of passengers. Concerning the identification of elements that characterise the road condition, besides potholes and other surface anomalies, speed bumps have also been identified as health hazards [6].

In this context, providing road condition information to different stakeholders is an important task for driver safety, convenience and comfort. This may be achieved by constantly surveying road surface for anomalies and undertaking corrective measures accordingly, such as repairing of roads or informing the stakeholders. Road pavement quality survey and anomalies detection is a useful service for various entities and different types of users. It is of interest to drivers of private as well as public transport vehicles. It is also important for public roads repair departments or other government authorities responsible for keeping the roads safe and in good condition.

The traditional methods for surveying the road condition are either by using specialised vehicles that have laser sensors or by surveying roads manually. However, these methods are very time consuming and expensive, especially while maintaining a large network of roads in big cities [7]. As an alternative, an emergent sensing paradigm, known as Collaborative Mobile Sensing or Mobile Crowdsourcing, is being discussed as a promising mechanism for collecting large-scale real-world data [8]. Collaborative mobile sensing is a people-centred method of sensing, in which participants collect and contribute with sensing data, using mobile devices such as smartphones [9]. The advantage of using smartphones is that it enables the participation of many contributors through crowdsourcing, thus enriching the system with a variety of data [10].

Road anomaly detection or road condition survey systems based on collaborative mobile sensing typically detect and automatically classify road anomalies using data-mining approaches on data collected by smartphones [11,12,13,14,15]. This is a challenging task, especially when the data are collected in real-world deployments. The use of smartphones imposes several problems and limitations when compared with more expensive and specialised road-monitoring equipment. Specifically, smartphones use less expensive sensors that typically offer less precision when compared to specialised equipment. This is especially true for inertial and GPS sensors.

This heterogeneity is not only related to smartphones and sensors. The automotive park suffers from the same problems, with many different car makers and models, and distinct suspension types and tyres. Furthermore, driving speed has a considerable impact on vehicle vibration, creating higher vibration amplitudes in accelerometer data [16].

This work was carried out in the context of an industry–academia joint research and innovation project that aims at building road pavement quality services for drivers and government agencies. The objective was to evaluate a road anomalies detection system based on the data-mining approach described in a previous work [17], in a real world deployment, i.e., an environment in which data are collected during normal driving experience, which may originate some situations that were not perceived at the system design time. The data-mining approach in [17] was based on the CRISP-DM framework [18,19] that comprises the following phases: Business Understanding, Data Understanding, Data Preparation, Modelling, Evaluation and Deployment. Smartphones were placed inside a car and a data collection application transmitted the data obtained from the smartphone sensors to a cloud-based platform, while the car was being driven. These “raw” data were then processed using various techniques to extract relevant features and machine-learning algorithms were applied for road anomalies recognition. Modelling and evaluation phases were based on a data set acquired in a “conditioned” setup, in which vehicles were moving on previously chosen road segments, with controlled speed.

Our real-world deployment evaluation approach consisted of two phases. In the first phase, we analysed the system’s performance in terms of the correct recognition of road anomalies in the real world deployment and compared the results with results obtained during the evaluation in the “conditioned” setup [17]. We have found that there was a difference in the system’s performance, where the results obtained in the real world deployment were worse than the ones obtained in the pervious evaluation phase.

In the second phase, we aimed at identifying some issues that may have influenced the system’s performance. The objective was threefold. First, in the context of the algorithm’s training model, we intended to evaluate if training data sets that included sample generated from data collected through smartphones in the real world deployment would provide more robust models.

Second, we analysed the complexity of the data as resulted from the data preparation phase of CRISP-DM framework. In particular, we executed some experiments to find out whether reducing or transforming data features would enhance the system performance. We based our features analysis on the Principal Component Analysis (PCA) technique [20].

Third, considering the differences between the system’s performance in each case, observed in the first phase, we provided an attribute-based analysis in the context of each anomaly type to better understand the characteristics of the acquired data and how those characteristics may influence in the distinction of different classes of anomalies.

The paper is structured as follows. A discussion on related work in the above domains is presented in Section 2. Section 3 briefly describes the approach for the road anomalies detection system, already presented in [17]. Section 4 gives details on the deployment of the system, and the underlying services architecture. Section 5 presents the results of the first phase of the evaluation. Section 6 presents a training model analysis. Section 7 provides an analysis of the selected attributes/features set. Section 8 presents an attribute-based analysis in the context of each anomaly type. The conclusions are discussed in Section 9.

## 2. Related Work

There are standardised measures to calculate and describe the road quality and condition. For example, the pavement condition index (PCI) is commonly used to describe the extent of distress on a pavement section [21] while International Roughness Index (IRI) is a statistic commonly used to estimate the amount of road roughness in a measured longitudinal profile. Both indices are calculated from pavement condition data parameters.

One cost effective approach to collecting road data is using smartphone sensors. There have been several works on estimating the road roughness and pavement quality by using smartphones sensors to collect the road surface vibration data.

Two comprehensive surveys review and discuss various road conditions detection systems found in existent literature [22,23]. According to these reviews, typically, related works apply machine-learning or threshold based methods on data acquired from smartphones’ accelerometer sensors to detect potholes, cracks, speed bumps, or other road anomalies [11,14,24,25,26,27,28,29,30,31]. Alternatively, other smartphone-based solutions have used microphones to record sound signals induced by potholes [26] or cameras for pothole detection [32]. Concerning the detection methods, it has been shown that, in addition to algorithms for road condition monitoring that rely on thresholding techniques, we may also find machine-learning based algorithms such as linear predictive coding, Support Vector Machines, k-means clustering, decision-tree classifiers or Bayesian networks [22].

Most of the work in this area has however been on experimental basis, considering data acquired in a limited and controlled manner. Two studies close to our work are presented in [14,28] where the smartphones were used to collect driving data, in a more realistic setting, to calculate the IRI or the road roughness condition. However, the focus of our work is the detection and the identification of road anomalies. The work presented in [24] also showed good results while detecting road anomalies, especially potholes, by using mobile phone sensor data acquired by a taxi network.

In [31], the authors approached the evaluation of a set of seminal heuristics that have guided and influenced the development of new anomaly detection strategies. For this aim, they provided a web platform that can be freely used by the community to create reliable and challenging datasets to perform their own experiments. They also described and evaluated a new method for road anomaly detection based on a Support Vector Machine enriched with novel features inspired in existent works, which has been proven to produce competitive results. Our work differs from those presented in the way that we mainly focus on the analysis of the issues that may impact the overall performance of a road anomalies detection system, in terms of the training model, data features selection and influence of the characteristics of acquired data in the distinction of different classes of anomalies.

A road condition anomalies detection system is a data centred system involving acquisition and processing many data. Data need to be transformed to be more comprehensive, increasing their usability in data-mining algorithms. In particular, data features must be defined and calculated. For example, in the context of data acquisition from accelerometer sensors, Nomura and Shiraishi [10] calculated vertical acceleration variances, Mukherjee and Majhi [33] used acceleration means, and Bello Salau et al. [12] used standard deviation of accelerometer data and presented a new measure called “z-difference square”, which uses the square of z-axis magnitude (Z0-Z1) to get only positive values.

The evaluation of feature extraction is a complex issue. It is usually associated with the evaluation of the data-mining approach itself. One approach to the analysis of a feature set is to apply the process of dimensionality reduction in the cases where some features may mislead the machine learning algorithm. A technique used for this purpose is called Principal Component Analysis (PCA) [20], which is an orthogonal transformation of possibly correlated variables (features) into independent variables, called principal components, through a statistical method. This technique has been widely used in various areas, including in medical research. For example, Weaver and Huddleston [34] used it for examining X-ray images. Abdullah et al. [35] could increase the accuracy of MRI brain classification by reducing the feature vector’s size using PCA. A more detailed survey on the application of PCA for medical image processing was given in [36]. In [37], PCA has been used in the context of anomaly detection where a big amount of complex data with high dimensionality and highly correlated variables was available. In the present work, we applied this technique to analyse the complexity of the data features set and in which extent it could influence the system performance.

In the context of road anomalies detection, there are some factors that may affect the system’s ability to detect and recognise road anomalies correctly from the data acquired from smartphone sensors while driving. There are two types of factors involved here: human and hardware factors. An example of a human factor is the driver’s behaviour, which has been analysed in [38]. Hardware factors, such as sensitivities of different smartphones, were highlighted in [14]. In the presented work, we analysed the ability of standard deviation to distinguish road anomalies.

## 3. Road Anomalies Detection Approach

We adopted a data-mining approach to detect and recognise road anomalies. This approach was previously described in detail in [17]. We followed the CRISP-DM framework [18,19] that comprises the following steps: Business Understanding, Data Understanding, Data Preparation, Modelling, Evaluation and Deployment.

The Business Understanding step consisted of the study of the problem domain and its specificities. It involves defining what we intend to predict, how it is characterised and the detailed definition of the system’s goal. Considering the project’s global objective of detecting and recognising road anomalies using data-mining approaches on data collected by smartphones and the current state-of-the-art, we first defined the set of common anomalies in our geographical area (Braga, Portugal). At first, we considered Potholes, Manholes and Bumps. However, the class of Potholes was eliminated, due to the low number of samples. The class of Bumps was divided in two classes: Short Bumps and Long Bumps. This was made due to the perceived differences on driving distress caused by these two types of bumps. Normally, Long Bumps are present in cross walk zones. These bumps are bigger and higher than Short Bumps, forcing drivers to significantly decrease the vehicle speed. We have also created an additional class that includes all the anomalies that do not fit in the previous classes. This class was named as “Others”. As a result of the Business Understanding step, the problem was defined as classifying areas on roads as Manholes, Short Bumps, Long Bumps, Others or No Anomaly (i.e., points without any anomaly detected).

**Figure 1:**
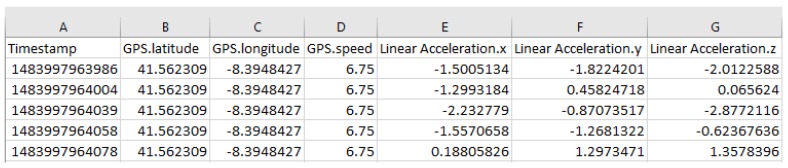
Collected data sample.

The Data Understanding step consisted of the analysis of data characteristics, such as data types, data values range and other specificities and associated problems. Having adopted the Collaborative Mobile Sensing paradigm through the use of smartphones, we analysed the available smartphone sensors to choose which data to collect. We chose the accelerometer and GPS sensors. From accelerometer sensor, we obtained acceleration values in the three axes (X, Y, and Z). X-axis corresponds to lateral acceleration, Y-axis corresponds to vertical acceleration and Z-axis corresponds to longitudinal acceleration. From the GPS sensor, we obtained the GPS coordinates and speed of the smartphone, allowing us to compute the location of road anomalies, in the following phases. The acquired data were also associated with a timestamp. Acceleration, GPS coordinates and speed are numeric, decimal values that can be positive or negative. Timestamp is an integer and positive value. A sample of the collected data is presented in Figure 1.

For data acquisition and establishing the ground truth, we developed an Android application that reads data from the accelerometer sensor at the rate of 50 Hz and reads GPS data at the rate of 1 Hz. Anomaly labelling was done simultaneously with data acquisition. For this propose, there were two persons in the vehicle, one driving and the other doing labelling. In the system’s design phase, data acquisition and labelling was done in a “conditioned” setup, in which vehicles were moving on previously chosen road segments, with controlled speed. This was done to prepare the initial dataset with known labels for training purposes. At the end of data acquisition, data samples were manually processed to correct labelling information to eliminate errors introduced when in movement. The data acquisition and labelling phases concluded with 157 anomalies recorded, divided into four classes.

The Data Preparation step consisted of the data cleaning and features extraction processes. From the raw data values, 50 features were extracted by performing calculations over a window of a fixed size (125 rows in our case). These attributes are values such as “maximum”, “mean”, “standard deviation”, “square difference” on each of the set of 125 values, for all the three accelerometer axes, X, Y and Z, separately. As anomalies may spread out over a window, we implemented a sliding window strategy with 60% overlap. This overlap was created to deal with windows that cut the anomaly into two or more parts. With the overlap we try to have, at least, one window that could represent the entire anomaly. The final features selection was done using WEKA (Waikato Environment for Knowledge Analysis) [39]. This process selected 25 attributes out of the 50 originally calculated attributes.

The Modelling step consisted of the selection and training of machine learning algorithms for road anomalies detection and recognition. We applied different supervised learning based algorithms [40]. For training the algorithms, we used 66% of the data set. After the training phase, we tested the different algorithms using the remaining 34% of the data set. To evaluate the algorithms, we chose score, accuracy, precision and confusion matrix measures.

The Evaluation step consisted of the analysis of the obtained results when recognising anomalies types, using the previously defined measures. During our analysis with various machine learning algorithms, Random Forest [41] was the algorithm that showed the best results.

The final step of the CRISP-DM framework refers to the deployment of the data-mining model in the real world. This deployment and its evaluation are the subjects of the following sections.

## 4. Road Anomaly Service

With the objective of evaluating the anomaly detection model described in Section 3 with data collected in real-world conditions, we designed, developed and deployed a *cloud-based road condition/anomaly information management* service based on vehicle context data, collected during driving activities using smartphones, and a Web application for identifying road anomalies. The overall functional process is as follows. Smartphones collect inertial data, i.e., accelerometer data for X, Y, and Z axes, GPS coordinates, speed, and bearing (this attribute is not used for anomaly detection), during driving activity. For this, smartphones are mounted on the vehicle’s windshield in a vertical portrait orientation. Data are periodically sent to the cloud infrastructure, where they are cleaned, processed and classified using the approach described in Section 3 for identifying road pavement anomalies according to their types. The Road Anomaly service manages and provides access to the resulting road anomaly information, which associates GPS coordinates with the respective road pavement condition, i.e., anomaly type.

### 4.1. System Architecture

**Figure 2:**
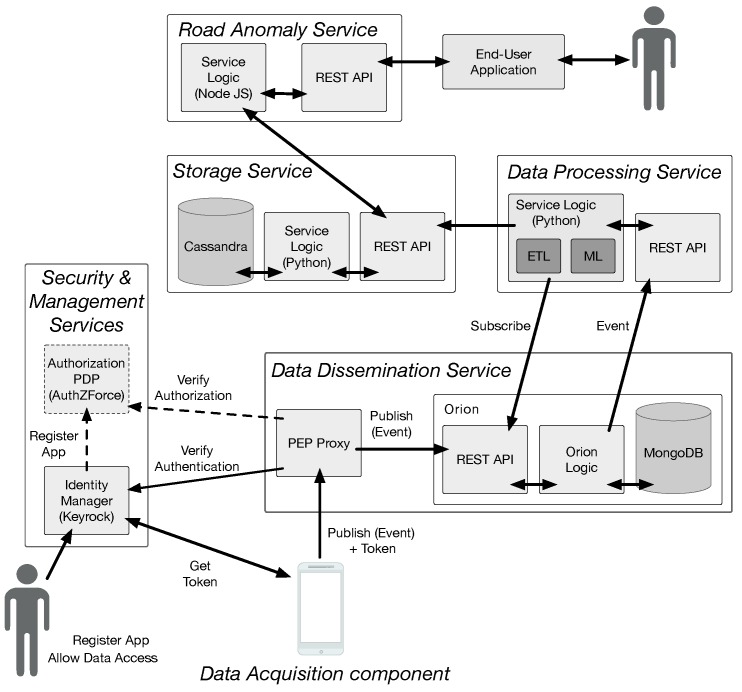
Road Anomaly Service architecture.

The overall system architecture is depicted in Figure 2. This is a cloud-based architecture, where each system component is designed, developed and deployed as an independent micro-service [42].

The *data acquisition* component is an Android native application that is responsible for acquiring sensor data during driving activity. This application starts data acquisition whenever the user is driving and smartphone is in portrait orientation and in a vertical position. If such conditions change, data acquisition is stopped. To detect the user’s activity, we used PathSense activity recognition library (PathSense’s site URL: https://pathsense.com/awesomeactivity). Energy management and cost management were also addressed in this component, as it prevents data transfers over mobile network connections (3G or LTE), in favor of Wi-Fi network connections. Acquired data are temporarily stored on the smartphone’s filesystem and sent to the *data dissemination component* when an active Wi-Fi connection becomes available.

The *data dissemination* service decouples component interactions, offering increased fault-resilience and scalability. This service was implemented using Fiware’s Orion Context Broker (CB) component (Fiware’s Orion CB site URL: https://catalogue.fiware.org/enablers/publishsubscribe-context-broker-orion-context-broker). Orion CB is a publish/subscribe broker, designed and developed in the context of the FIWARE project [43].

The *data processing* service performs data cleaning, data transformation, and classification for each request. Each request consists of fixed size datasets with 1 Mbyte of dimension. This partition was performed by the data acquisition component, during the acquisition process. Each request is processed concurrently by a separate process. Service scalability is achieved by horizontally scaling the service with the number of requests, thus increasing the number of available instances.

The *storage* service is responsible for managing information, by storing information resulting from the data processing service and offering other services/applications access to it. This service must support data management functionalities, offering scalable information storage, while maintaining its performance. Our current implementation is backed by the Cassandra database management system (Cassandra’s site URL: http://cassandra.apache.org). Cassandra is designed to handle high data volumes, while offering scalable performance and, if necessary, high availability, both of which are achieved by increasing the number of instances.

The *road anomaly* service consumes data from the storage service and provides a higher level abstraction of this information to other components. In this particular case, it provides: road anomaly information in a given bounded area, for example, a rectangular area defined by the coordinates of the top left and bottom right corners; road anomaly information of user-defined types; and, detailed information of user-defined anomalies.

Finally, a set of orthogonal services, namely, the Identity Manager service, the Authorization Policy Decision Point (PDP) service, and the Policy Enforcement Point (PEP) Proxy, deal with some security and privacy issues. The Identity Manager allows developers to register applications and IoT devices, and manage data access policies. It provides the foundations for authenticating IoT devices (or data producing applications), enforcing security policies. In conjunction with the Authorization PDP, it also enforces privacy policies, managing authorizations for applications to access data. The PEP Proxy works in conjunction with both these services, for validating authentication and authorization policies, guaranteeing that only authenticated and authorized applications can publish or have access to data. These services were implemented using Fiware’s Identity Manager, Keyrock, Authorization PDP, and PEP Proxy.

### 4.2. Road Anomaly Identification Application

**Figure 3:**
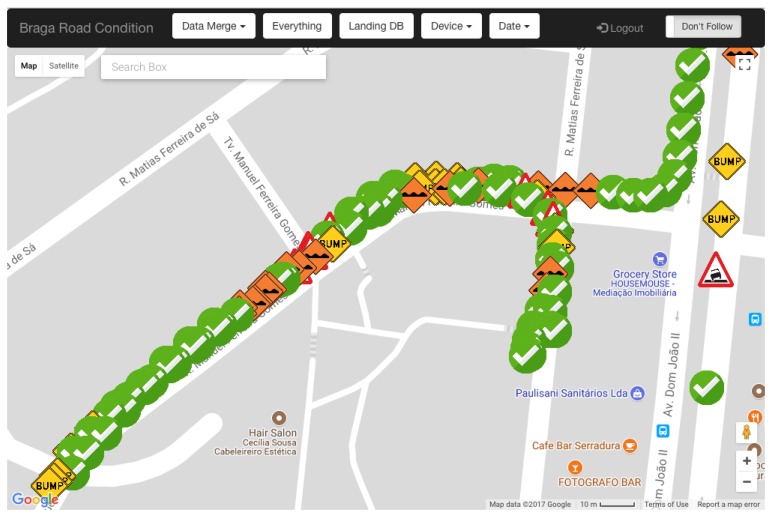
Road anomaly identification application.

The Road Anomaly Identification Web application was designed as a tool to visually inspect and to manually identify road anomaly information. To this end, it allows users to visualise aggregated and non-aggregated road anomaly information on top of a city street map, with and without applying map-matching techniques. Anomalies can be then manually validated according to their actual type. This allows us to collect physical anomaly information and to use it to evaluate the performance of the anomaly detection model in real-world conditions. Figure 3 presents a view of the Road Anomaly Validation application. Geo-referenced markers represent different road anomaly types, providing visual differentiation of road anomalies.

**Figure 4:**
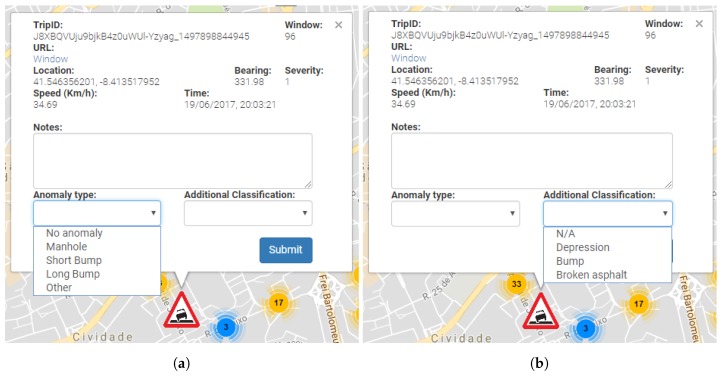
Anomaly validation: (**a**) anomaly type validation; and (**b**) additional anomaly information.

Anomalies may be manually identified, as described in Figure 4a,b. This identification process allows detected anomalies to be manually identified according to the actual anomaly type (Figure 4a) and also allows additional information to be added (Figure 4b). While this additional information is not currently being used, it may provide useful information in future iterations of the project.

## 5. Road Anomalies Detection System Evaluation

The deployment evaluation of the road anomalies detection system consisted of the analysis of the system’s performance in terms of the correct recognition of road anomalies by comparing the current results with the results obtained during the previous evaluation phase [17].

### 5.1. Evaluation Process Specification

**Figure 5:**
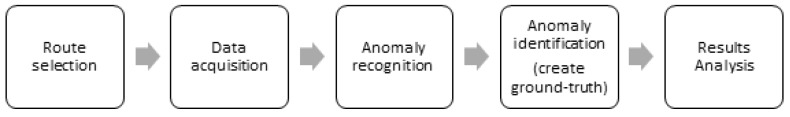
Evaluation process.

We defined an evaluation process that is depicted in Figure 5. This process consists of five steps: Route selection; Data acquisition; Anomaly recognition; Anomaly identification; and Results analysis.

**Figure 6:**
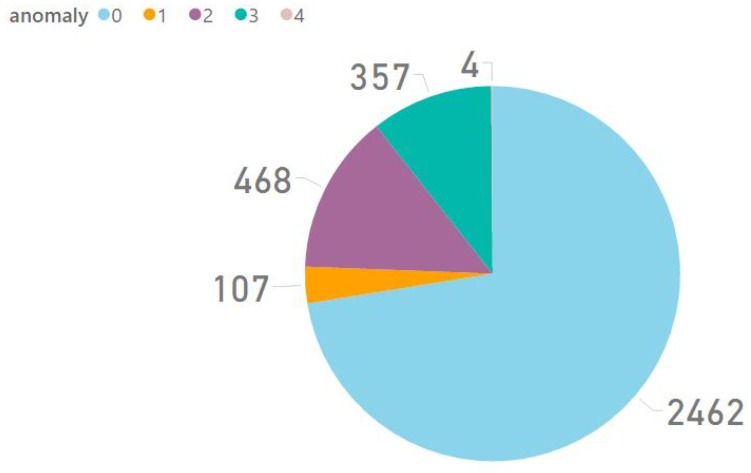
Anomalies recognised by the system.

We selected some roads in Braga and Guimarães, two cities in the north of Portugal, comprising a total of 40 km of roads. Data acquisition was performed using three cars—a Toyota Avensis, a Hyundai Accent and a Peugeot 308 SW—equipped with one smartphone each—a Samsung Galaxy S4 Mini (i9195) and two LG Nexus 5—mounted on the iOttie Easy One Touch 3 (https://www.iottie.com/Product/Detail/2118/iOttie-Easy-One-Touch-3-Car-Mount-for-iPhone- Smartphones) holder. Smartphones ran the Android data acquisition application presented in Section 4. Driving activity was performed without any constraints of speed or road design. Anomaly recognition was then performed by the data processing service and road anomalies information was provided to consumers, such as drivers or government entities, by the service providing layer as described in Section 4. In total, the system recognised approximately 3400 samples, distributed as depicted in Figure 6, where anomaly type 0 corresponds to “No Anomalies”, type 1 to “Manhole”, type 2 “Short Bump”, type 3 to “Long Bump” and type 4 to “Others”.

**Figure 7:**
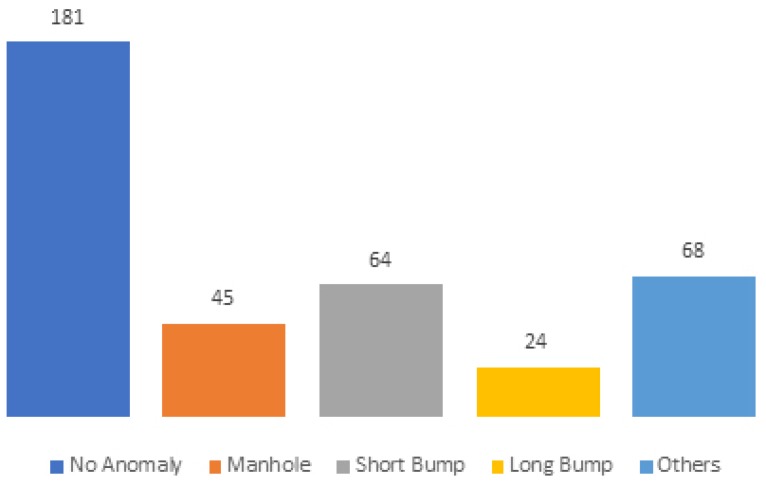
Anomalies with real label.

Anomaly identification is essential as a basis for establishing the ground-truth for the system performance evaluation. Anomaly identification was performed using the anomaly identification application described in Section 4. In total, we identified 382 anomalies distributed over anomaly classes, as depicted in Figure 7.

### 5.2. Analysis of Results

To evaluate the system performance we have calculated the following measures: Score (*True Positives/Total Samples*) , Precision (*True Positives*/(*True Positives + False Positives*)) and Accuracy ((*True Positives + True Negatives*)/(*Total number of instances*)). The values of these measures were then compared with the corresponding values of the previous evaluation (in “conditioned” setup [17]) as presented in Table 1. We also calculated the Score for each anomaly class (Table 2). Finally, we also calculated the confusion matrices to analyse how the system behaves for each anomaly class (Table 3 and Table 4). The confusion matrix shows how actual anomalies were recognised by the system.

On the one hand, comparing the results in Table 1, it is evident that there is a significant decrease in values of each of the chosen measures. On the other hand, analysing Table 2, Table 3 and Table 4, we can perceive that the recognition score of the class “No Anomaly” is similar in both settings.

Although the system in the real world setting has achieved a good score in recognising “No Anomalies” (probably due to the low values in accelerometer readings that are very distinguishable from the anomalies high values), the overall score would compromise the service level quality as the system also intended to distinguish anomalies classes in the real world. Further analysis of the results and of the whole process is necessary. In the following sections, we describe our results on the analysis of three different issues that may influence the system performance. Firstly, we focus on the analysis of the training data sets. Secondly, we analyse the complexity of the data set attributes. Thirdly, we analyse the attributes in the context of each anomaly type in order to evaluate how the recognition process may be improved.

## 6. Model Training Analysis

In the context of machine learning algorithms, exhaustive data sets used as training samples tend to improve system’s accuracy. In our contextd, this is particularly important to recognise variations of a road anomaly. While selecting an exhaustive data set for training, caution is needed, however, so that there is neither over-fitting (by hand-picking a few “perfect”-looking examples for each anomaly class) nor over-generalisation (by creating a sample that would represent almost everything). Our hypothesis was that training data sets including samples generated from smartphone data collected in the real world deployment help training a more robust model. This is because such data sets may include more diverse data for the same anomaly class, which may reflect real conditions. To verify our hypothesis, we designed a new model. The model was trained and tested using the data collected in the real world deployment. We used 66% of the data set for training and 34% of the data set for testing. We applied the “Random Forest” algorithm.

Score, accuracy and precision were calculated. Table 5 and Table 6 present the score, accuracy and precision measures, as well as the confusion matrix, in the three different settings (for Settings 1 and 2, we used the values presented in Section 5). Setting 1 shows the results using “Controlled” data in training and testing; Setting 2 used “Controlled” data for training and “Uncontrolled” to testing; and Setting 3 used “Uncontrolled” data for testing and training.

The score in Setting 3 is higher than the one achieved in Setting 2, and slightly lower than the one achieved in Setting 1. In addition, the precision value of Setting 3 is significantly higher than that of Setting 1. These results point out that training the model with data acquired in a real world setting may improve the robustness of the model. Particularly, considering the precision evaluation, there is a great probability of identifying anomalies when they actually exist, reducing the chances of false positives.

## 7. Feature Set Analysis

In the context of machine learning algorithms, feature extraction is a complex issue, as selected features may mislead the machine learning algorithm. In this section, we describe the results of applying the Principal Component Analysis (PCA) technique [20] for reducing and transforming the initial set of features that has been applied in our first evaluation. PCA is a technique used to decrease the features set dimensionality. This is accomplished by calculating new features (components) using normalised linear combination of the original ones and considering their variances, i.e., considering their effectiveness in pattern recognition.

For this evaluation, the PCA was applied to the original 25 dimensional feature set. First, the PCA implementation process is described and then the results are evaluated.

### 7.1. PCA Application Process

**Figure 8:**
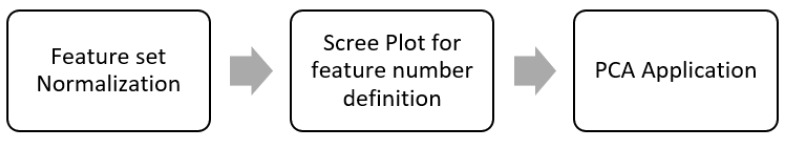
PCA implementation process.

Figure 8 depicts the PCA implementation process that comprises three phases. The first phase consisted of the normalisation of the original 25-dimensional feature set. The process of normalisation involves transforming all attributes in way that the mean is equal to zero and standard deviation is equal to one.

**Figure 9:**
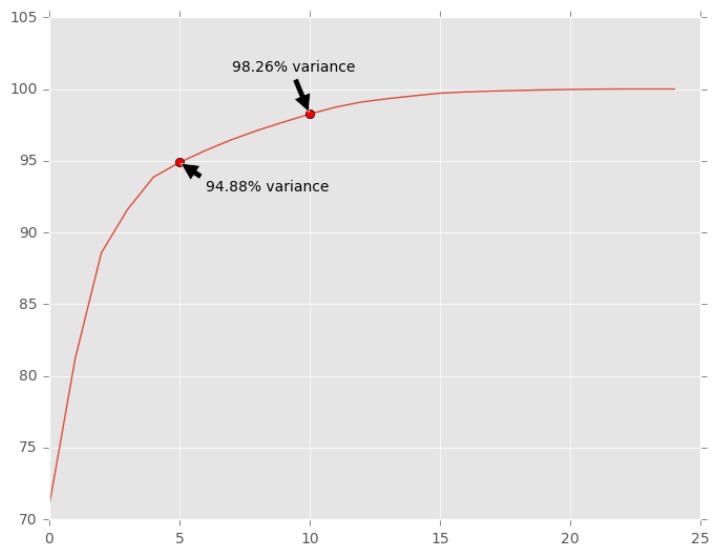
Scree plot showing components variance cumulative sum.

The second phase consisted of the definition of the number of features as input to the PCA application process. This was achieved through the creation of a Scree Plot (Figure 9). A Scree Plot shows the cumulative proportion of variance. From this Scree Plot, we may observe that variances of 95% and 98% may be achieved with 5 and 10 features, respectively. It could then be concluded that, when reducing the number of features to 5 and 10, only 5% and 10% of variance would be lost, respectively. Thus, 5 and 10 features were then defined for the PCA application.

Finally, the third phase consisted of the application of the two PCA analyses. These processes generated two sets of features (PCA components) and the corresponding datasets.

### 7.2. PCA Results Analyses

To analyse the results of the PCA application, we trained and tested the machine learning model in the same conditions as described in Section 5: the model was trained with data collected in the “conditioned” setup and tested with data collected in the real world deployment. The Score, Precision and Accuracy metrics for each model are presented in Table 7. Comparing both models, we may observe that their performance is similar. In addition, comparing both models with the deployed model (with results presented in Section 5), we may observe that there are no significant differences in performance. The results of the deployed model are slightly superior though.

Considering the confusion matrices presented in Table 7, we may also observe that there are not relevant differences between the two models that might have resulted from PCA application and between those and the previously deployed model.

Considering the above results, we may say that reducing and transforming model features would not enhance the system performance.

## 8. Anomaly Attributes Analysis

The evaluation of the anomaly recognition model in a real world deployment, discussed in Section 5, has shown that there was a difference in the system’s performance when compared with the system’s performance in a “conditioned” setup, mainly in the aspect of distinguishing types (classes) of anomalies. To better understand the characteristics of the acquired data, the current section provides an attribute-based analysis in the context of each anomaly type. Our main goal is to find some indicators that may contribute in improving the anomaly recognition model in following iterations of the system deployment, focusing mainly on the problem of distinguishing different classes of anomalies.

The presented analysis focuses on the attributes generated from the X and Y axes of the accelerometer because road anomalies, typically, have greater impact in the values of the vertical accelerometer axis (accelerometer Y-axis when smartphone is in vertical position). Additionally, one way to differentiate road anomalies types is through detecting the distinct effects observed on the accelerometer’s X axis. We chose the attribute standard deviation, as, in a first analysis, this attribute was found to be more effective in detecting and recognising anomaly classes. Hence, the graphics in this section show the interactions between acceleration standard deviation X and acceleration standard deviation Y for the instances of the designated classes.

In each of the analyses presented below, the graphics show anomaly instances as validated by ground-truth. The first analysis is between samples classified as “No anomaly” and samples classified as “Anomaly” (any anomaly class). This analysis shows the aforementioned attributes’ effectiveness in detecting the anomaly (and “no anomaly”) cases. Thereafter, the subsequent analyses are between different anomaly classes to analyse the complexities involved in correctly identifying individual anomaly classes, especially when pairs of classes are overlapping on each other in the attributes space.

**Figure 10:**
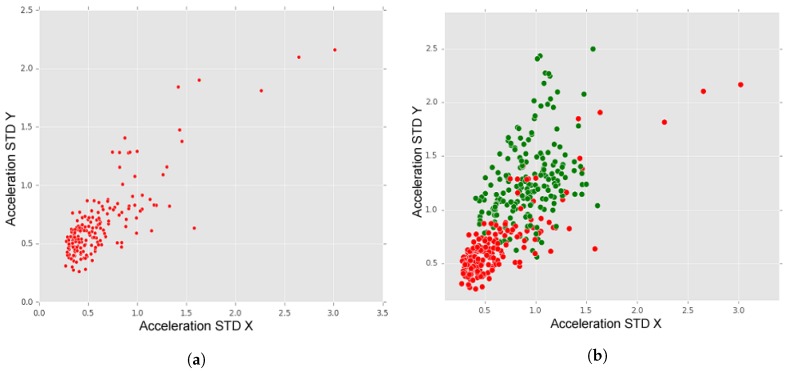
(**a**) No anomaly; and (**b**) no anomaly (red dots) and anomalies (green dots).

In Figure 10a, we may observe the dispersion of “no anomaly” instances and, in Figure 10b, we may observe a comparison between the dispersion of “no Anomaly” instances (red dots) and instances of all types of anomalies (green dots). In Figure 10a, although we may observe some outliers, it is visible that “no anomaly” instances are situated between zero and one (in both axes).

**Figure 11:**
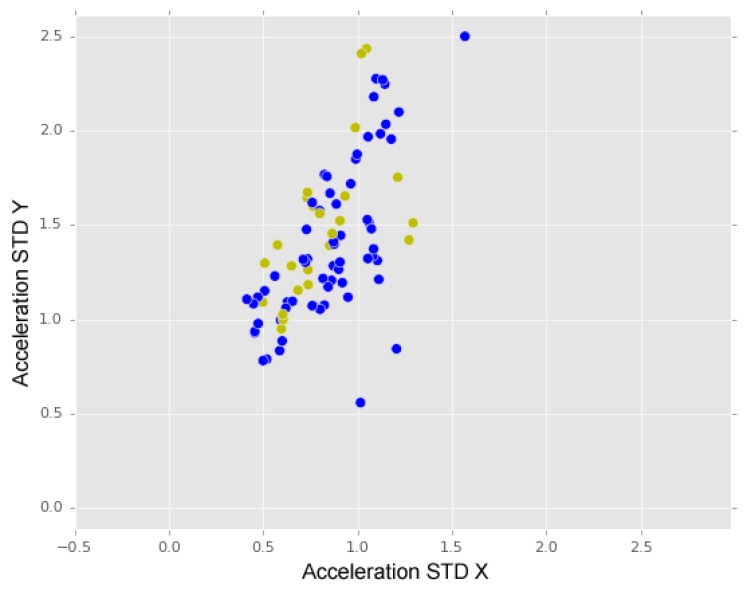
Long bumps (yellow dots) and short bumps (blue dots).

In Figure 10b, the difference between “no anomalies” and “anomalies” is visible. The green dots represent anomalies instances (“manholes, “short bumps”, “long bumps” and “others”), which present higher values of acceleration standard deviation, when compared with “no Anomaly” data. In Figure 11, we may observe a comparison between the dispersion of “short bumps” instances (blue dots) and “long bumps” instances (yellow dots). From this representation, we may observe that “short bumps” and “long bumps” have similar values for acceleration standard deviation for X and Y axes, which may indicate that a higher accuracy rate may be achieved if these two anomalies classes are merged into one class.

**Figure 12:**
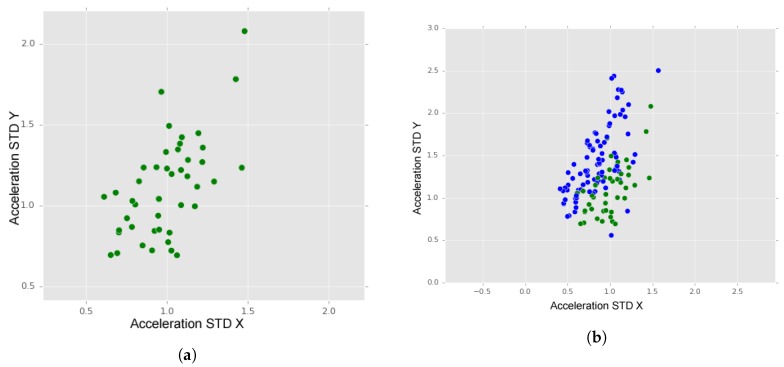
(**a**) Manholes; and (**b**) manholes (green dots) and bumps (blue dots).

In Figure 12a, we may observe the dispersion of “manholes” instances (green dots). As it is visible, the dispersion of manholes data along both axes shows a similarity between values of X and Y accelerations.

Considering this information and projecting “manholes” instances and the instances of the two types of “bumps” in the same graphic (Figure 12b), we may observe that these two classes are distinguishable. Although the separation is not total, we may observe higher values on Y axis for bumps data than for manholes data, thus distinguishing the two.

**Figure 13:**
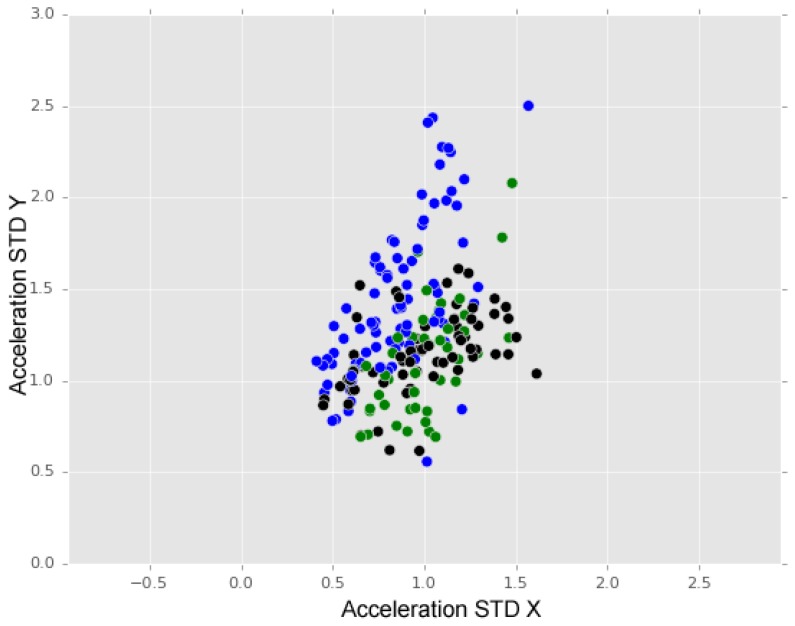
Bumps (blue dots), manholes (green dots), and others (black dots).

To finalise our analysis, we analysed the effects of introducing the anomaly class known as “others”. The introduction of the class “others” in the training process may have influenced the overall training if the associated attribute values were similar to the ones of the specific anomaly types. Figure 13 shows a distribution of “others” instances (black dots) compared with the “bumps” (blue dots) and “manholes” (green dots) instances distributions. We may observe that instances of class “others” are spread over instances of the other two classes.

**Figure 14:**
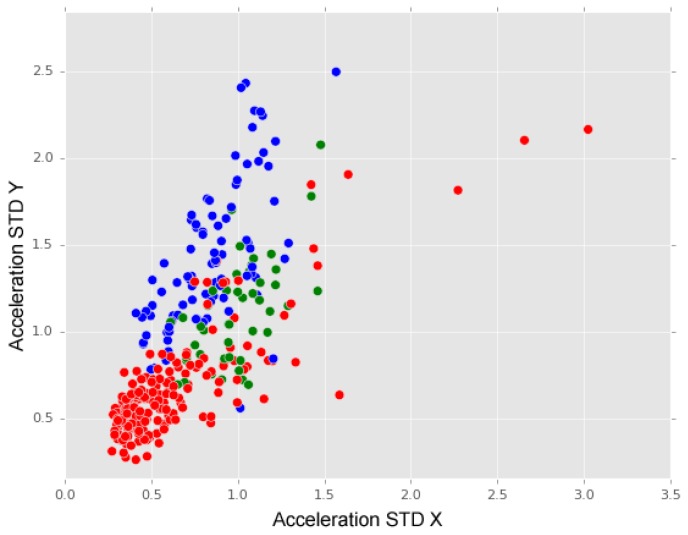
No anomalies (red dots), major anomalies (blue dots) and minor anomalies (green dots).

Considering the above analysis and observed impacts of anomalies on X and Y acceleration axes, we argue that the subsequent iterations of the anomaly recognition model should consider an alternative classification of anomalies. Figure 14 represents a distribution of anomalies in which “long bumps” and “short bumps” classes are merged into a new class named “major Anomaly” (blue dots), i.e. anomalies that a driver is more likely to cross over, and “manholes” class has given origin to a new class named “minor anomaly” (green dots)— anomalies that a driver may avoid. In this graphic, we may observe that these two classes of anomalies are distinguished by their differences on Y-acceleration axis values. Complementary, “no anomaly” instances are characterised by small values registered on both X and Y acceleration axes.

The design of a new iteration of the anomaly recognition model must also consider a reclassification of “others” class because, as we have shown in the above analysis, “others” instances spread over both “long and short bumps” and “manholes” instances.

## 9. Conclusions

Anomalies on road pavement cause discomfort to drivers and passengers, and may cause mechanical failure or even accidents. Governments spend millions of Euros every year in road maintenance, often causing traffic jams and congestion on urban roads on a daily basis.

In this paper, we describe a road anomalies detection system based on collaborative mobile sensing that detects and automatically classifies road anomalies using data-mining approaches on data collected by smartphone sensors, more specifically accelerometer data. We also presente a service-oriented architecture that supports data acquisition, data processing, data storage, a road anomalies service and the development of end-user applications in the context of road condition domains. We deployed the road anomalies detection system in a real-world environment, in which driving activity was performed without any constraints of speed or road design. This was achieved using an end-user application for validating anomalies information, building a new ground-truth to evaluate the model’s performance. In total, 382 events were validated.

Score, precision and accuracy results in the real world deployment are significantly lower than score, precision and accuracy results in the “conditioned” setup. This represents a decrease in the system’s performance in the real world, particularly in recognising road anomalies and in distinguishing road anomalies classes. In a subsequent analysis of the training data sets, when including the data acquired in a real-world setup in the training phase, the overall performance (Score measure) of the system did not improve. Nevertheless, we can observe an improvement in system’s precision. This may be because real-world data sets may include bigger diversity of data for the same anomaly class, which may reflect real conditions. This result points out that work on characterisation and standardisation of the data acquisition process for real anomaly detection is needed. Similarly, our attempt to reduce the complexity of the data set attributes through PCA techniques has not shown any improvement of the final results.

When focusing on the aspect of distinguishing types (classes) of anomalies, we produced a visual analysis of the interactions between acceleration standard deviation X and acceleration standard deviation Y for the instances of the chosen anomalies classes. In this analyses, the aforementioned attributes’ effectiveness in detecting the anomaly (and “no anomaly”) cases and some similarity between the aforementioned attributes’ values for “long” and “short bumps” classes were observed. This may indicate that the classification problem should be divided into two main steps: first detecting the existence of an anomaly and then identifying its class. Furthermore, current results may show that long and short bumps classes should be merged into a unique class.

Taking all these factors into consideration, the deployment of our system helped us in gaining more insights about road anomalies detection challenges. This work is the first iteration of an on-going development towards the deployment a road anomaly detection service to provide information about roads condition to drivers and government entities. Despite the differences in system’s performance between “conditioned” and real-world setups, we have obtained some insights that should support the design and deployment of a new iteration of our system.

## Figures and Tables

**Table 1:** Comparison between “conditioned” setup and real world deployment.

Metrics	Laboratory	Real World
Score	0.8674	0.5628
Precision	0.7521	0.3623
Accuracy	0.9469	0.8251

**Table 2:** Score per class.

Anomaly Classes	Score in Laboratory	Score in Real World
No Anomaly	0.96	0.94
Manhole	0.50	0.20
Short Bump	0.81	0.48
Long Bump	0.78	0.13
Others	0.56	0

**Table 3:** Confusion Matrix for “Condtitioned” Setup.

	Detected As				
	No Anomaly	Manhole	Short Bump	Long Bump	Others
No Anomaly	342	4	10	1	1
Manhole	16	26	2	5	3
Short Bump	5	0	25	1	0
Long Bump	5	4	3	42	0
Others	1	4	2	1	10

**Table 4:** Confusion matrix for real world deployments.

	Detected As				
	No Anomaly	Manhole	Short Bump	Long Bump	Others
No Anomaly	171	0	5	5	0
Manhole	16	9	17	3	0
Short Bump	23	1	31	9	0
Long Bump	9	1	10	4	0
Others	29	3	22	14	0

**Table 5:** Confusion Matrices.

			Setting 1					Setting 2					Setting 3				
			Recognised Anomaly Class														
			No Anomaly (0)	Manhole (1)	Short Bump (2)	Long Bump (3)	Other (4)	No Anomaly (0)	Manhole (1)	Short Bump (2)	Long Bump (3)	Other (4)	No Anomaly (0)	Manhole (1)	Short Bump (2)	Long Bump (3)	Other (4)
**Confusion** **Matrix**	**Actual** **Anomaly** **Class**	No Anomaly (0)	342	4	10	1	1	55	0	0	0	1	171	0	5	5	0
		Manhole (1)	16	26	2	5	3	4	10	0	0	1	16	9	17	3	0
		Short Bump (2)	5	0	25	1	0	2	0	12	0	4	23	1	31	9	0
		Long Bump (3)	5	4	3	42	0	0	0	3	1	3	9	1	10	4	0
		Other (4)	1	4	2	1	10	0	0	0	0	19	29	3	22	14	0
Score			0.8674					0.5628					0.8434				

**Table 6:** Metrics.

		Setting 1		Setting 2		Setting 3	
Training Dataset		Controlled		Controlled		Uncontrolled	
Testing Dataset		Controlled		Uncontrolled		Uncontrolled	
		Accuracy	Precision	Accuracy	Precision	Accuracy	Precision
**Anomaly** **Class**	No Anomaly	0.9162	0.9268	0.7723	0.6895	0.9391	0.9016
	Manhole	0.9259	0.6842	0.8927	0.6429	0.9565	1
	Short Bump	0.9552	0.5952	0.7723	0.3647	0.9217	0.8
	Long Bump	0.9610	0.84	0.8665	0.1143	0.9478	1
	Other	0.9766	0.7143	0.8219	0	0.9217	0.6786
**Overall**			0.7521		0.3623		0.8760

**Table 7:** PCA Confusion Matrices.

			PCA-5 Features					PCA-10 Features				
			Recognised Anomaly Class									
			No Anomaly (0)	Manhole (1)	Short Bump (2)	Long Bump (3)	Other (4)	No Anomaly (0)	Manhole (1)	Short Bump (2)	Long Bump (3)	Other (4)
**Confusion** **Matrix**	**Actual** **Anomaly** **Class**	No Anomaly (0)	172	3	2	3	1	173	1	2	4	1
		Manhole (1)	22	9	2	7	5	28	11	2	2	2
		Short Bump (2)	23	4	21	16	0	29	2	17	16	0
		Long Bump (3)	10	0	5	7	2	10	1	3	10	0
		Other(4)	35	12	6	13	2	41	7	5	14	1
**Score**			0.5524					0.5550				
**Precision**			0.3827					0.4339				
**Accuracy**			0.8209					0.8220				

## Abbreviations

The following abbreviations are used in this manuscript:

CB	Orion Context Broker
CRISP-DM	Cross Industry Standard Process for Data Mining
GPS	Global Positioning System
IoT	Internet of Things
IRI	International Roughness Index
LTE	Long-Term Evolution
MRI	Magnetic resonance imaging
PCA	Principal Component Analysis
PCI	Pavement Condition Index
PDP	Policy Decision Point
PEP	Policy Enforcement Point
STD	Standard Deviation
WEKA	Waikato Environment for Knowledge Analysis
